# Brain immune interactions and air pollution: macrophage inhibitory factor (MIF), prion cellular protein (PrP^C^), Interleukin-6 (IL-6), interleukin 1 receptor antagonist (IL-1Ra), and interleukin-2 (IL-2) in cerebrospinal fluid and MIF in serum differentiate urban children exposed to severe vs. low air pollution

**DOI:** 10.3389/fnins.2013.00183

**Published:** 2013-10-10

**Authors:** Lilian Calderón-Garcidueñas, Janet V. Cross, Maricela Franco-Lira, Mariana Aragón-Flores, Michael Kavanaugh, Ricardo Torres-Jardón, Chih-kai Chao, Charles Thompson, Jing Chang, Hongtu Zhu, Amedeo D'Angiulli

**Affiliations:** ^1^Department of Biomedical Sciences, The Center for Structural and Functional Neurosciences, The University of MontanaMissoula, MT, USA; ^2^Hospital Central Militar, Secretaria de la Defensa NacionalMexico City, Mexico; ^3^Department of Pathology, School of Medicine, University of VirginiaCharlottesville, VA, USA; ^4^Centro de Ciencias de la Atmósfera, Universidad Nacional Autónoma de MéxicoMexico City, Mexico; ^5^Department of Biostatistics, Gillings School of Global Public Health, University of North CarolinaChapel Hill, NC, USA; ^6^Department of Neuroscience, Carleton UniversityOttawa, ON, Canada

**Keywords:** Alzheimer, air pollution, children, innate immunity, neurodegeneration, neuroinflammation, particulate matter, prion cellular protein

## Abstract

Mexico City Metropolitan Area children chronically exposed to high concentrations of air pollutants exhibit an early brain imbalance in genes involved in oxidative stress, inflammation, innate and adaptive immune responses along with accumulation of misfolded proteins observed in the early stages of Alzheimer and Parkinson's diseases. A complex modulation of serum cytokines and chemokines influences children's brain structural and gray/white matter volumetric responses to air pollution. The search for biomarkers associating systemic and CNS inflammation to brain growth and cognitive deficits in the short term and neurodegeneration in the long-term is our principal aim. We explored and compared a profile of cytokines, chemokines (Multiplexing LASER Bead Technology) and Cellular prion protein (PrP^C^) in normal cerebro-spinal-fluid (CSF) of urban children with high vs. low air pollution exposures. PrP^C^ and macrophage inhibitory factor (MIF) were also measured in serum. Samples from 139 children ages 11.91 ± 4.2 years were measured. Highly exposed children exhibited significant increases in CSF MIF (*p* = 0.002), IL6 (*p* = 0.006), IL1ra (*p* = 0.014), IL-2 (*p* = 0.04), and PrP^C^ (*p* = 0.039) vs. controls. MIF serum concentrations were higher in exposed children (*p* = 0.009). Our results suggest CSF as a MIF, IL6, IL1Ra, IL-2, and PrP^C^ compartment that can possibly differentiate air pollution exposures in children. MIF, a key neuro-immune mediator, is a potential biomarker bridge to identify children with CNS inflammation. Fine tuning of immune-to-brain communication is crucial to neural networks appropriate functioning, thus the short and long term effects of systemic inflammation and dysregulated neural immune responses are of deep concern for millions of exposed children. Defining the linkage and the health consequences of the brain / immune system interactions in the developing brain chronically exposed to air pollutants ought to be of pressing importance for public health.

## Introduction

Air pollution is a significant health problem in megacities around the world (Molina and Molina, 2004; Chen and Kan, 2008; Bloom, 2011). In the first 50 years of this century (Bloom, 2011), the projected world population will have a further increase of 2–4.5 billion making the issue of deteriorating environments and their health impact critical.

Mexico City Metropolitan Area (MCMA) children with no known risk factors for neurological or cognitive disorders exhibit significant deficits in a combination of fluid and crystallized cognition tasks vs. low air pollution exposed children (Calderón-Garcidueñas et al., 2008a). Brain structural and volumetric changes are seen in both MCMA children and young animal facility dogs (Calderón-Garcidueñas et al., 2008a, 2011a, 2012a). The canine frontal white matter lesions are characterized by vascular sub-cortical pathology associated with neuroinflammation, gliosis, and ultrafine particulate matter deposition (Calderón-Garcidueñas et al., 2008a). Young MCMA residents exhibit the neuropathological hallmarks of Alzheimer and Parkinson's diseases i.e., amyloid beta42 (Aβ42) plaques, tau hyperphosphorylation with pre-tangles and α-synuclein accumulation (Calderón-Garcidueñas et al., 2012b).

The complex modulation of cytokines and chemokines that influence children's central nervous system (CNS) structural and volumetric responses to air pollution obligate the search for biomarkers associating systemic and CNS inflammation to brain growth and cognitive deficits in the short term and neurodegeneration in the long-term (Calderón-Garcidueñas et al., 2008a,b, 2011a,b, 2012b). A good candidate biomarker, macrophage migration inhibitory factor (MIF) is a cytokine expressed in the CNS and involved in innate and adaptive immune responses (Bernhagen et al., 1993; Bacher et al., 2010; Edwards et al., 2010; Moon et al., 2012; Savaskan et al., 2012; Bucala, 2013; Cox et al., 2013; Freiria-Oliveira et al., 2013; Turtzo et al., 2013). MIF participates in the induction of neural stem/progenitor cells (Ohta et al., 2012), the protection of mice female brains after experimental stroke (Turtzo et al., 2013), the mediation of the antidepressant action of exercise (Moon et al., 2012), and of key importance for this work, MIF plays a key role in neuroinflammatory responses (Savaskan et al., 2012; Cox et al., 2013).

In clinical practice and in the experimental setting, MIF has been used as a marker of neurological worsening in progressive neurodegenerative processes including multiple sclerosis and autoimmune-mediated neuroinflammation (Hagman et al., 2011; Cox et al., 2013), as a key neuro-immune mediator linking depressive symptoms with inflammation and HPA dysregulation (Edwards et al., 2010), and as a predictor of lack of response to antidepressants (Cattaneo et al., 2013). Moreover, in the scenario of air pollution and the early hallmarks of Alzheimer's disease (AD) in Mexico City children (Calderón-Garcidueñas et al., 2008a, 2012a), two key issues are of utmost interest to us: the increased concentrations of MIF in the cerebro-spinal-fluid (CSF) of AD patients and the capacity of MIF to enhance the ability of the tau/Aβ42 ratio to discriminate cognitively normal vs. mildly demented patients (Lee et al., 2008; Bacher et al., 2010; Craig-Schapiro et al., 2011). In addition MIF promotes the production of several inflammatory mediators including IL-6, TNF α, and IFN γ (Popp et al., 2009). Given the wide spectrum of serum and CSF responses to pathological processes including neuroinflammation and neurodegenerative diseases such as AD and Parkinson's disease (PD) (Dziedzic, 2006; Helmy et al., 2012; Yan et al., 2012; Giralt et al., 2013; Martinez et al., 2013; Mooijaart et al., 2013; Smolen et al., 2013), we have selected a 41 cytokine/chemokine panel and MIF to explore the CSF responses to high air pollution residency.

Cellular prion protein (PrP^C^) is a conserved GPI-anchored membrane protein located on the surface of neurons, at both pre and post-synaptic sites, with a high abundance in the hippocampus, frontal cortex and stratium (Stellato et al., 2011; Ding et al., 2013; Watt et al., 2013). The PRNP gene located in chromosome 20 likely plays key roles in neuronal development, synaptic plasticity, myelin sheath maintenance, cell adhesion, CNS development, and neuroprotection via inhibition of the mitochondrial apoptotic pathway (Mitsios et al., 2007; Altmeppen et al., 2012; Bradford and Mabbott, 2012; Kaiser et al., 2012; Ding et al., 2013).

We are particularly interested in the critical role PrP^C^ may play in the integrity of the CNS under a neuroinflammatory insult, precisely the key marker of air pollution exposure in urban children (Bremer et al., 2010; Popko, 2010; Gourdain et al., 2012; Scalabrino and Veber, 2012). The role of PrP^C^ in cellular immunity (Tsutsui et al., 2008), cognitive functioning (Breitling et al., 2012), oxidative stress, and the impact of prion proteins upon the toxic effect of environmental neurotoxic metals (Choi et al., 2007; Oh et al., 2012) are at the core of our research.

The primary aim of this study was to measure the concentrations of PrP^C^ and selected inflammatory mediators in normal CSF from cohorts of children with high exposures to urban air pollution vs. low pollution control children. Concurrently, we also explored serum MIF and PrP^C^ in clinically healthy children representing cohorts with high and low air pollution exposures.

Our results identify CSF as a MIF, IL6, IL-1Ra, IL-2, and PrP^C^ compartment that differentiates children with high vs. low air pollution exposures. The MIF increases in CSF and serum compartments in highly vs. low exposed children strongly suggest MIF could be a serum biomarker bridge to identify children at risk for brain inflammation. Short and long term effects of dysregulated neural immune responses and systemic inflammation are of pressing importance for public health.

## Procedure

### Study areas

Children's cohorts were selected from the MCMA and several small cities in Mexico, characterized by clean environments with concentrations of the six criteria air pollutants (ozone, particulate matter, sulfur dioxide, nitrogen oxides, carbon monoxide and lead) below the current US EPA standards.

### Participants

This research was approved by the research ethics committee of the Hospital Central Militar in Mexico City. Children gave active assent and their parents gave written informed consent to participation in the study. This work includes data from 139 children 74F, 65M (*Meanage* = 11.91 years, *SD* = 4.2), carefully selected to represent comparable populations from a larger longitudinal cohort research program. There were two groups of children included in this study: Group 1 (*n*: 28, *Meanage* = 10.46 years, *SD* = 4.2, low pollution 8F/6M; high pollution 6F/8M) corresponded to children admitted to the hospital from either MCMA or a low polluted city with a work up diagnosis of acute lymphoblastic leukemia entering a clinical protocol, which included a spinal tap. Only cases with a normal CSF were included in this study. None of the selected CSF samples belonged to children with previous oncological and/or hematological treatments. Group 2 (*n*: 111, 44 controls (24F/20M), 67 MCMA (33F/34M), *Meanage* = 13.37 years, *SD* = 4.2) were clinically healthy children from MCMA and control cities and their serum samples were taken as part of their pediatric examination during a longitudinal follow-up. Inclusion criteria for all participating children were: negative smoking history and environmental tobacco exposure, lifelong residency in MCMA or a control city, residency within 5 miles of the city monitoring stations, full term birth, and unremarkable clinical histories prior to the hospital admission (for the children CSF donors). Low and high pollution exposed children were matched by age, gender, and socioeconomic status.

### CSF samples

Spinal tap was performed in the supine position from lumbar levels using a standard 22 spinal needle. CSF was collected dripping in free air in1 ml aliquot into Nalge Nunc polypropylene CryoTubes. Lumbar puncture samples were collected during non-traumatic, non-complicated procedures. CSF pleocytosis was defined as CSF white blood cell (WBC) counts of >7 cells per mm^3^. CSF samples were used for the laboratory procedures without dilution steps.

### Peripheral blood analysis

Blood was collected from an antecubital vein using a 21-G needle. After centrifugation at 3000 rpm for 10 min, aliquots of 1.5 ml serum were transferred to CryoTubes and samples were frozen at −20°C and then transferred to −80°C and stored until further analysis. Blood samples were also collected for a complete blood count (CBC) with differential. Serum samples were processed for Multiplexing LASER Bead Technology (Eve Technologies, Calgary, Canada) that included 41 cytokines and chemokines: EGF, Eotaxin, FGF-2, Flt-3L, Fractalkine, G-CSF, GM-CSF, GRO, IFNα2, IFNγ, IL-1α, IL-1β, IL-1ra, IL-2, IL-3, IL-4, IL-5, IL-6, IL-7, IL-8, IL-9, IL-10, IL-12 (p40), IL-12 (p70), IL-13, IL-15, IL-17a, IP-10, MCP-1, MCP-3, MDC, MIP-1α, MIP-1β, PDGF-AA, PDGF-AB/BB, RANTES, sCD40L, TGFα, TNFα, TNFβ, and VEGF. An ELISA was done for the quantification of PrPc in serum and CSF, BetaPrion®Human EIA (AJ RoboScreen GmbH, Leipzig, Germany). Macrophage inhibitory factor (MIF) was done using an ELISA procedure from R&D (Minneapolis, MN 55413, USA). Serum samples were run without dilution steps.

### Data analysis

First, we calculated the summary statistics (mean ± standard deviation) of all relevant variables including age, inflammatory mediators and PrPc results. In the 28 CSF samples, we worked with 42 variables and established with independent samples *t*-tests which variables were statistically significant between children with high exposures to air pollutants (Mexico City) and children residing in low pollution cities (Controls). Spearman correlations were run between the CSF target inflammatory mediators and PrPc. In the 111 serum samples, we worked with 2 variables and established with independent samples *t*-tests which variables were statistically significant between Mexico City and control children. Significance was assumed at *p* < 0.05 and data expressed as mean values ± SD. All the statistical analyses described above were performed on statistical software “R” (http://www.r-project.org/).

## Results

### CSF data

CSF samples were clear, colorless, with a normal opening pressure, a mean WBC count of 2.1 ± 1 cells per mm^3^ and no RBC. Glucose was 55.9 ± 6.7 mg/100 ml in controls and 54.8 ± 11.7 mg/100ml in Mexico City children (*p* = 0.3). Table 1 shows the results of the 42 variables included in the analysis. Six variables were statistically significant, including: MIF, PrP^C^, IL-6, IL1Ra, IL-2, and MIP1α (this is not an effect of the number of multiple comparisons, i.e., inflation of Type I error, because the binomial probability that 6 comparisons out of 42 would turn out to be significant by chance is less than 0.0001). Relevant Spearman correlations between CSF PrP^C^ and target inflammatory mediators are seen in Table 2. We found significant correlations between PrP^C^ and IL-15 (*p* = 0.025) and RANTES (*p* = 0.04), MIF and IL-6 (*p* < 0.000) and IL-6 with IL-15 and RANTES (*p* = 0.03).

**Table 1:** **Cerebro-spinal- fluid inflammatory mediators in Control vs. Mexico City children (values are shown in pg/ml)**.

**Variables**	**Mean (controls)**	***SD* (controls)**	**Mean (Mexico City)**	***SD* (Mexico City)**	***p*-value**
EGF	1.627	2.258	2.416	2.665	0.396
FGF2	11.972	20.495	13.294	12.858	0.838
TGFalpha	2.810	1.637	1.800	1.667	0.111
GCSF	8.389	3.668	10.602	12.673	0.526
FlT.3L	23.429	18.261	35.249	34.856	0.261
GM.CSF	4.327	2.253	2.907	1.500	0.060
Fractalkine	41.711	18.529	218.851	422.998	0.127
IFNα2	4.931	2.468	5.349	2.989	0.683
IFNγ	0.278	0.148	1.296	2.006	0.070
GRO	8.057	13.421	19.084	22.972	0.125
IL10	0.259	0.258	2.593	4.966	0.091
MCP3	3.574	3.358	3.594	5.018	0.990
IL12p40	2.030	1.131	2.526	1.564	0.335
MDC	16.996	6.852	20.768	11.320	0.285
IL12	0.473	0.939	0.637	0.516	0.571
PDGF.AA	4.351	4.690	3.210	3.483	0.467
IL13	0.516	0.229	0.477	0.255	0.673
PDGF-BB	0.963	1.494	0.635	1.301	0.535
IL15	2.920	2.491	5.237	5.317	0.144
sCD40l	2.391	5.617	1.334	1.547	0.507
IL17a	5.165	18.595	0.225	0.021	0.338
IL1ra	0.475	0.579	1.915	1.954	0.014
IL1a	0.323	0.365	0.905	1.329	0.122
IL9	0.996	1.229	0.952	0.861	0.913
IL1beta	0.231	0.043	0.642	1.389	0.272
IL2	0.218	0.042	0.530	0.532	0.040
IL3	0.220	0	0.709	1.929	0.343
IL4	2.099	0.890	2.788	1.627	0.167
IL5	0.534	0.385	3.769	12.514	0.334
IL6	1.161	0.650	21.26	38.5	0.006
IL7	0.251	0.080	0.429	0.445	0.147
IL8	17.306	26.099	26.341	37.853	0.459
IP10	207.479	492.590	296.127	519.441	0.641
MCP1	1061.199	1159.217	1521.123	2698.525	0.553
MIP1α	1.130	1.846	6.497	9.129	0.041
MIP1β	6.776	3.065	7.049	3.761	0.832
RANTES	2.121	1.720	8.667	13.963	0.093
TNFα	0.368	0.224	1.441	3.0208	0.191
TNFβ	0.471	0.843	0.987	2.384	0.442
VEGF	4.542	4.494	24.503	43.563	0.099
PrPc	4.396	3.228	8.036	5.506	0.039
MIF	66.918	24.454	329.930	264.342	0.002

**Table 2:** **Spearman correlations in CSF target inflammatory mediators and PrP^C^ Control v Mexico City children**.

**Mediator**	**PrP^C^*r*/*p*-value**	**MIF *r*/*p*-value**	**IL-6 *r*/*p*-value**	**IL-15 *r*/*p*-value**	**RANTES *r*/*p***
PrP^C^	1000	0.307 /0.113	0.236/0.302	0.424/0.025	0.406/0.044
MIF	0.307/0.113	1000	0.773/0.000	0.233/0.232	0.236/0.256
IL-6	0.236/0.302	0.773/0.000	1000	0.467/0.033	0.476/0.034
IL-15	0.424/0.025	0.233/0.232	0.467/0.033	1000	0.692/0.000
RANTES	0.406/0.044	0.236/0.256	0.476/0.034	0.692/0.000	1000

### Serum data

MIF and PrP^C^ results in serum are seen in Table 3. MIF was significantly higher (*p* = 0.009) in Mexico City children, while PrP^C^ showed no differences between cohorts.

**Table 3:** **Serum MIF and PrP^C^ in Control v Mexico City children**.

**Variable**	**Controls**	**Mexico City**	***p*-value**
MIF	425.1 ± 188	711.1 ± 488.7	0.009
PrP^C^	1.671 ± 0.96	1.647 ± 1.465	0.21

*Values are shown in pg/ml*.

## Discussion

Cerebro-spinal-fluid concentrations of Macrophage migration inhibitory factor MIF, IL-6, IL-1Ra, IL-2, Macrophage Inflammatory Protein 1-α [MIP1α,Chemokine (C-C Motif) Ligand 3] and PrP^C^ differentiate children with high vs. low air pollution exposures and suggest a complex interplay of a network of multipotent cytokines and normal cellular proteins with known neuromodulatory actions participating in neuroinflammatory responses associated with exposures to air pollutants. Our data also suggests that MIF in serum is a potential biomarker bridge to identify children with CNS inflammation.

IL-6, IL-1Ra, IL-2, and MIF are important players in the pathogenesis of neuroinflammation and the systemic inflammation associated with chronic exposures to high concentrations of air pollutants, while the increased CSF concentrations of PrP^C^ could potentially represent a neuroprotective response against oxidative stress (Griffiths et al., 2007; Carnini et al., 2010).

IL-6 is a pleiotropic cytokine with key roles in inflammatory responses and in directing T cell differentiation in adaptive immunity. The elevation of IL-6 in the CSF of highly exposed children and its significant correlation with other potent chemokines and cytokines regulating and coordinating innate and adaptive immune responses is important in the developing CNS.

IL-6 has been implicated in systemic and brain neo-angiogenesis (Gertz et al., 2012). In traumatic brain injury the presence of low concentrations of IL-6 [IL-6 knock-out (KO) mice] is associated with poor behavior performance, and impacts the expression of IL1β, another powerful pro-inflammatory cytokine (Ley et al., 2011). Neuroprotective associations of specific IL-6 SNP (i.e., the G-allele of the SNP rs1800795) on hippocampal volumes (Baune et al., 2012), the protection of midbrain dopaminergic neurons by IL-6 in the model of 1-methyl-4-phenyl-1,2,3,6-tetrahydropyridine (MPTP) (Spittau et al., 2012), and the significant attenuation of IL-6 concentrations and lower gray volume in old rhesus macaques by a caloric-restricted diet (Willette et al., 2010) support a complex interaction between IL-6, other inflammatory mediators and protective brain responses. The role of IL-6 in long-term potentiation (LTP) and learning is well known (Del Rey et al., 2013). The increased expression of IL-6 is causally related to an increase in synaptic strength since it was abrogated when LTP was interfered by blockade of NMDA-glutamate receptors (Del Rey et al., 2013).

Detrimental effects on the other hand, are described between high concentrations of IL-6, reduced hippocampal volumes and major depressive disorders (Frodl et al., 2012) and in chronic systemic inflammatory conditions with increased risk of stroke (Drake et al., 2011). In advanced HIV infection, CSF concentrations of IL-6 show elevations in patients on suppressive combination antiretroviral therapy regardless of cognitive status, implying the persistence of intra-thecal inflammation even in the absence of clinical manifestations (Kamat et al., 2012).

Of utmost importance for this work and the higher concentrations of CSF IL-6 in Mexico City children is the fact we are seeing the hallmarks of Alzheimer i.e., hyperphosphorilated tau and Aβ42 amyloid plaques in 44 and 51%, respectively, of highly exposed Mexico City teens vs. 0% in low pollution control children. In the literature involving elderly individuals, the associations between IL-6 and dementia are controversial (Papassotiropoulos et al., 2001; Jellinger, 2010; Helmy et al., 2012). Galimberti et al., reported the highest CSF IL-6 concentrations in AD patients with mild cognitive deterioration, suggesting to the authors a key role of IL-6 in the initial phases of neurodegeneration (Galimberti et al., 2008). Combarros et al., suggested that dysregulation of IL-6 in some elderly people contributes to the development of AD (Combarros et al., 2009). Oxidative damage and neuroinflammation are crucial players in neurodegenerative diseases (Nunomura et al., 2001, 2006, 2012a,b), and both are present in the context of air pollution exposures (Calderón-Garcidueñas et al., 2012a). Moreover, under experimental conditions, exposures to different components of air pollutants cause oxidative stress, neuroinflammation and neurodegeneration (Calderón-Garcidueñas et al., 2003, 2008a, 2012a,b; Levesque et al., 2011, 2013). Thus, the CSF and brain inflammatory imbalance observed in highly exposed children could represent an early physiological reaction to chronic environmental stress contributing later to the establishment of neurodegenerative processes with childhood clinical manifestations (Calderón-Garcidueñas et al., 2008b, 2011b, 2012b, 2013). Of critical importance are the portals of entry of air pollutants in the urban setting: (i) the nasal pathway through the olfactory, trigeminal nerves and accessory posterolateral nerve, (ii) the red blood cells and monocytes transporting ultrafine PM and then delivering the particles to distant organs including the brain, (iii) the direct access of PM organic and inorganic components to the systemic circulation through the alveolar-capillary interphase, (iv) the gastrointestinal and vagal pathways (Calderón-Garcidueñas et al., 2007; Block and Calderón-Garcidueñas, 2009; Dhuria et al., 2010; Bleier et al., 2012; Lucchini et al., 2012).

Thus, the association between IL-6 serum increases and deficits in verbal memory (Grassi-Oliveira et al., 2011) obligates us to explore the cognitive consequences of an inflammatory imbalance and cognitive impairment in highly exposed cohorts (Aung et al., 2011; Tsai et al., 2012; Uski et al., 2012; Wittkopp et al., 2013).

Intrathecal inflammation in these children is counterbalanced by the increased production of interleukin 1 receptor antagonist (IL-1Ra), a natural endogenous antagonist of IL-1, critical for a variety of brain effects (Arend, 2002; Gadek-Michalska and Bugajski, 2010; Akash et al., 2013; Peters et al., 2013). IL-1RA exhibits low uptake in the normal brain, with rapid metabolism, a short biological half-life (4–6 h), and excretion via the kidneys (Cawthorne et al., 2011). IL-1Ra higher CSF concentrations in highly exposed children, is a key observation since we have previously described a significant frontal up-regulation of IL-1β in Mexico City children (Calderón-Garcidueñas et al., 2008a). IL-1Ra has clear neuroprotective effects in experimental perinatal inflammation and hypoxic-ischemic injuries (Girard et al., 2012), reduces ischemic brain damage and inflammation in co-morbid rats (Pradillo et al., 2012), prevents postoperative cognitive decline and neuroinflammation in older rats (Barrientos et al., 2012), and plays a key anti-inflammatory role in AD (Rubio-Perez and Morillas-Ruiz, 2012). Interestingly, experimental endotoxemia in healthy male volunteers produces significant transient increases in IL-1Ra and decreases in mood, likely reflecting a compensatory strategy or a greater social cognitive processing as a function of sickness (Kullmann et al., 2013). We interpreted the CSF IL-1Ra increases as a neuroprotective response in Mexico City children.

On the other hand, the increases in CSF IL-2 are in keeping with the critical role of this pro-inflammatory cytokine in both effector T-cell development and FoxP3(+) CD4(+) Treg-cell homeostasis (Shameli et al., 2013). Of utmost interest in the context of air pollution is the role of IL-2 in regulating inflammation in an organ-specific manner through the migration and retention of CD4(+) T-cells (both Th1 and Th2) (Ju et al., 2012). Since IL-2 is also required for regulating the Th2 cytokine response during T-cell activation and participates in multifactorial autoimmune responses (Wang et al., 2009; Baine et al., 2013), the issue of autoimmunity to CNS components warrants full investigation in exposed children especially those with extensive white matter hyperintense lesions (Calderón-Garcidueñas et al., 2008b, 2012b).

Macrophage Inflammatory Protein 1-α [Chemokine (C-C Motif) Ligand 3/CCR3] CSF increases in Mexico City children relate to its well known role in inflammatory responses, angiogenesis and tissue remodeling through binding with receptors to other chemokines (i.e., CCR1, CCR4, CCR5) (Gaspar et al., 2013).

MIF is a newly arrived player in the air pollution scenario, is an active participant in innate and acquired immunity (Savaskan et al., 2012), its association with the production of several inflammatory mediators including IL-6, TNF-α, and IFN-γ, and the identification of CSF MIF as a biomarker in AD and mild cognitive impairment patients (MCI) (Popp et al., 2009; Craig-Schapiro et al., 2011) has a great impact in air pollution-related brain effects.

The role of MIF as a neuro-immune mediator linking inflammation with depressive symptoms and HPA dysregulation (Anisman and Hayley, 2012; Savaskan et al., 2012), and its concomitant increases in CSF and serum makes MIF a potential biomarker to identify children with systemic and CNS inflammation. A facet of MIF of importance for us is the potentiation with autoimmune-mediated neuroinflammation (Cox et al., 2013). Cox et al. demonstrated that MIF is essential for microglial activation and the production of IL-6, IL-1β, TNF-α, and inducible NO synthase in an experimental autoimmune encephalomyelitis model. Cox et al., work is important to us because the target cytokines described in their paper are up-regulated in brain tissues of highly exposed children, and the same cytokines exhibit higher serum concentrations when compared with low pollution exposed children (Calderón-Garcidueñas et al., 2008a,b, 2009, 2011a, 2012a,b).

The CSF increases of the normal cellular isoform of the prion protein (PrP^C^) in exposed children deserve to be discussed under three important headings:

1. The PrP^C^ neuroprotective role related primarily to its antioxidant properties, its capacity as a radical scavenger, its key role in signal transduction and cell survival, neuronal zinc regulation, and its functional importance in the protection against oxidative stress and metal toxicity (Brown et al., 1997; Kuwahara et al., 1999; Viles et al., 1999; Milhavet and Lehmann, 2002; Hu et al., 2007; Westergard et al., 2007; Carnini et al., 2010; Martins et al., 2010; Bertuchi et al., 2012; Alfaidy et al., 2013; Watt et al., 2013).
2. The PrP^C^ role in animal behavior suggesting that the absence of the prion protein could result in altered neural processing (Massimino et al., 2013).
3. The associations of PrP^C^ with neuropathology apart from the conventional prion diseases (Jiménez-Huete et al., 1998; Ferrer et al., 2001; Voigtländer et al., 2001; Checler and Vincent, 2002; Aguzzi and Haass, 2003; Rezaie et al., 2005; Schwarze-Eicker et al., 2005; Ramljak et al., 2008). Of interest to us are the controversial issues in the literature linking Aβ oligomers and PrP^C^ (Laurén et al., 2009; Nygaard and Strittmatter, 2009; Gunther and Striitmatter, 2010; Kessels et al., 2010; Barry et al., 2011; Saijo et al., 2011; Larson et al., 2012; Um et al., 2012; Chen et al., 2013; Kudo et al., 2013; Whitehouse et al., 2013; Younan et al., 2013). A few specific examples to make the point: Kudo et al. showed that Prnp (-/-) mice are resistant to the neurotoxic effect of oligomeric Aβ *in vivo* and *in vitro* (Kudo et al., 2013). Chen et al. have shown PrP^C^ over-expression down-regulated tau protein and PrP^C^ lacking the Aβ oligomer binding site was incapable of rescuing the level of tau reduction. These authors demonstrated that PrP^C^ down-regulated tau via Fyn pathway and the effect can be regulated by Aβ oligomers (Chen et al., 2013). Whitehouse et al. measured PrP^C^ in the frontal cortex of 24 sporadic AD brains vs. 24 age-matched controls and found a significant decreased of PrP^C^ in AD brains. Interestingly, PrP^C^ significantly inversely correlated with BACE1 activity, Aβ load, soluble Aβ, and insoluble Aβ and with the stage of disease, as indicated by Braak tangle stage (Whitehouse et al., 2013). The authors concluded that brain PrP^C^ level may be important in influencing the onset and progression of sporadic AD (Whitehouse et al., 2013).

Why are all these papers relevant to our work? We recently published a paper (Calderón-Garcidueñas et al., 2012a) showing a 15 fold frontal down-regulation of mRNA PrP^C^ in Mexico City residents (18 ± 8.7 years) vs. low air pollution age matched controls. In the same work, we showed frontal tau hyperphosphorylation and Aβ diffuse plaques in the exposed kids, but none in low air pollution controls. Thus, the regional frontal PrP^C^ down-regulation shown by microarray analysis is of deep concern given the age of the autopsy subjects (on average 8 years older) than our CSF children in this study. PrP^C^ might have significant longitudinal variations in mRNA expression by brain region (Rezaie et al., 2005; Velayos et al., 2009), age, gender, and other factors in the setting of air pollution. Moreover, because the CSF is produced and reabsorbed throughout the entire CSF-Interstitial fluid (IF) functional unit (Chikly and Quaghebeur, 2013), our results highlight the need to define PrP^C^ changes both directly in different regions of the brain and in the CSF in low vs. high pollution exposed children and young adults. Furthermore, since PrP^C^ likely plays a key protective role against sporadic AD (Whitehouse et al., 2010; Griffiths et al., 2012) we will expect age-related decreases in specific regional brain areas inversely related to the development of AD hallmarks in highly exposed individuals.

The second reason for concern is the impact of PrP^C^ in cognition. Gimbel et al. showed that AD transgenic mice with intact PrP^C^ expression exhibit deficits in spatial learning and memory, while mice lacking PrP^C^, but containing Aβ plaque derived from APPswe/PSen1DeltaE9 transgenes, show no detectable impairment of spatial learning and memory (Gertz et al., 2010). Thus, deletion of PrP^C^ expression dissociates Aβ accumulation from behavioral impairment in AD mice, with the cognitive deficits selectively requiring PrP^C^ (Gertz et al., 2010). Interestingly, Schmidt et al. (2013) showed no association between CSF PrP^C^ and cognitive status in 114 AD patients, while Breitling et al. reported in a 5 years follow-up study of 1322 elderly Germans (aged 65+ years at baseline), an inverse association between serum PrPc and cognitive functioning (Breitling et al., 2012). In our study, CSF PrP^C^ was significantly higher in exposed vs. low pollution children, while serum PrP^C^ concentrations showed no differences.

The significant correlations of PrP^C^ with RANTES and IL-15 deserve a comment. Regulated upon Activation, Normal T-cell Expressed and Secreted (RANTES) is a proinflammatory chemokine produced by neurons and capable of upregulating neurotrophic factors and extending cell survival after ischemic stroke (Tokami et al., 2013), while IL-15 instructs the generation of a distinct memory T-lymphocyte subset, intermediate between naive and central memory cells (Cieri et al., 2013). These findings highlight the complex link between normal brain proteins, powerful chemokines, and immune driven cytokines.

It has been clear for a number of years that neuroinflammation has critical implications for cognition, behavior, altered brain growth and neurodegeneration. Brain dysbalances in inflammatory mediators and alterations of essential proteins with key roles in CNS development and neuroprotection will inevitably have detrimental consequences for young brains. We already described some of these consequences in Mexico City youth: differences in white matter volumes involving right parietal and bilateral temporal areas and cognitive deficits consistent with impairment of the targeted lobes, olfaction deficits, auditory and vestibular nuclei accumulating α synuclein and/or β amyloid and significant involvement of the medial superior olive neurons, critically involved in brainstem auditory evoked potentials. Elevation of indices of neuroinflammation and oxidative stress, and AD and PD associated pathology completing the grim picture (Calderón-Garcidueñas et al., 2003, 2008a,b, 2010, 2011a,b, 2012a,b, 2013; Calderón-Garcidueñas and Torres-Jardón, 2012).

At the core of our observations lies the complex dynamic interaction in different compartments of cytokines, chemokines and brain proteins responding to the constant state of inflammation and oxidative stress resulting from unhealthy environmental exposures. The short term consequences are having an impact in the performance of children in school and in their violent behavior (Calderón-Garcidueñas and Torres-Jardón, 2012), while the longer effects are already seen in neuropathological studies of children with accidental deaths (Calderón-Garcidueñas et al., 2008b, 2012b, 2013). There is no doubt of the close linkage between the brain and the immune system particularly in the developing brain (Bilbo and Schwarz, 2012). Brisk responses are at hand both systemically and in the brain upon endogenous and exogenous environmental signals, however, the developing brain is delicately sensitive to chronic stimuli and the end result could be a pathological process with deleterious outcomes for the individual.

## Looking forward and limitations

Despite controversy regarding the mechanistic pathways involved in the CNS damage associated with exposure to air pollutants, we agree that oxidative stress, endothelial dysfunction vascular damage and neuroinflammation are at the core of the pathology, and the same factors play a key role in neurodegenerative diseases (Qin et al., 2007; Rivest, 2009; Levesque et al., 2011; Castellani and Perry, 2012; de la Torre, 2012; Nunomura et al., 2012a,b; Calderón-Garcidueñas et al., 2013). We are looking forward to bridging the gap between early neuroinflammation and its neurodegenerative consequences, an issue of importance in childhood and adolescence when cognitive performance is critical.

There is a need for looking into the CSF early responses to air pollution because we can connect with the knowledge available for elderly populations, i.e., the progression of Alzheimer core biomarkers [Aβ(1-42), total tau and phosphorylated tau] and inflammatory markers useful in the clinical practice to evaluate patients with mild cognitive impairment and their course to AD. This is critical, given that implementation of early preventive pathways (Castellani and Perry, 2012) in the developing brain could ameliorate or drastically modified the pathology in later years.

Our results are potentially limited by the fact our CSF normal samples were taken from a population of children with a hematological workup for a neoplastic process, albeit no CNS involvement, while our serum samples were from clinically healthy children. As pediatricians we are totally aware of the ethical issues in taking CSF samples from healthy children, so the readily available normal CSF samples destined to be discarded are a good initial source of exploratory samples. Nevertheless, the robust CSF increase in the described inflammatory mediators in highly exposed children warrants extensive investigations using available normal CSF samples in Mexico and around the world. Two issues are important here: the selection of multiplex platforms and knowledge of their ability to accurately and sensitively detect cytokines in CSF and blood (serum/plasma) (Malekzadeh et al., 2012). The second one, the use of standardized operating procedures for the recollection of CSF samples, a key point if we want to decrease the variability attributed to different pre-analytical procedures between laboratories (del Campo et al., 2012).

## Summary

We have quantified CSF responses for a selected panel de inflammatory mediators and PrP^C^ in two groups of children with contrasting air pollution lifetime exposures. Our results identify MIF, IL6, IL1Ra, IL-2, and PrP^C^ as potential CSF biomarkers differentiating the high vs. low air pollution exposed children, supporting the previous evidence of an ongoing neuroinflammatory process in Mexico City children. Serum MIF was identified as a robust biomarker in Mexico City children.

Fine tuning of immune-to-brain communication is crucial to neural networks appropriate functioning, thus the short and long term effects of systemic inflammation and dysregulated neural immune responses are of deep concern for millions of highly exposed children.

A large body of work on PrP^c^, IL-6, MIF, IL-2, and the IL-1 family already exists, thus expanding this knowledge in the scenario of air pollution pediatric effects could greatly facilitate our understanding of the downstream mechanisms of the complex interaction of cytokines/chemokines/ PrP^c^ immune responses in the developing brain and the resulting short and long term brain effects.

The role of PrP^c^ in highly exposed children is an issue worth investigating further as the field moves forward, since we believe knowledge on PrP^c^ responses in a developing brain will shed light on neuroprotective mechanisms against the onset of Alzheimer disease.

Although the two objectives of this study were accomplished: (i) To evaluate the concentrations of PrP^C^ and selected inflammatory mediators in normal CSF from cohorts of children with high vs. low pollution controls and (ii) To explore serum PrP^C^ and MIF in clinically healthy children representing cohorts with high and low air pollution exposures, our immediate goal will be to assess whether the identified biomarkers will be useful in the identification of children at higher risk for cognitive deficits, behavioral disorders, and structural and volumetric brain alterations.

Our ultimate goal is that following the identification of children at the highest risk for detrimental brain effects, we protect them through multidimensional interventions yielding both impact and reach. The prospective of our efforts is readily apparent.

Defining the linkage and the health consequences of the brain/immune system interactions in the developing brain chronically exposed to air pollutants ought to be of pressing importance for public health.

## Author contributions

Lilian Calderón-Garcidueñas planned and directed the study, wrote the manuscript, performed all experiments and supported the study from personal funds. Janet V. Cross planned the study, performed the MIF determinations and wrote the manuscript. Amedeo D'Angiulli wrote, edited and formatted the manuscript and performed part of the statistic analysis. Maricela Franco-Lira and Mariana Aragón-Flores obtained patient consent and ethical approvals, got the samples and wrote the manuscript. Michael Kavanaugh wrote the manuscript. Chih-kai Chao and Charles Thompson performed the PrP^C^ and wrote the manuscript. Ricardo Torres-Jardón wrote the manuscript and Jing Chang and Hongtu Zhu performed the statistical analysis. All authors approved the final draft of the manuscript for publication.

### Conflict of interest statement

The authors declare that the research was conducted in the absence of any commercial or financial relationships that could be construed as a potential conflict of interest.
